# Oxidative stress in a cellular model of alcohol-related liver disease: protection using curcumin nanoformulations

**DOI:** 10.1038/s41598-025-91139-0

**Published:** 2025-03-05

**Authors:** Lucy Petagine, Mohammed G. Zariwala, Satyanarayana Somavarapu, Stefanie Ho Yi Chan, Evrim A. Kaya, Vinood B. Patel

**Affiliations:** 1 Centre for Nutraceuticals, School of Life Sciences, University of Westminster, 115 New Cavendish Street, London, W1W 6UW UK; 2 Department of Pharmaceutics, UCL School of Pharmacy, London, UK

**Keywords:** Alcohol, Antioxidants, Curcumin, Liver, Mitochondria, Oxidative stress, Reactive oxygen species, Biochemistry, Cell biology, Gastroenterology, Gastrointestinal diseases, Liver diseases, Alcoholic liver disease

## Abstract

Alcohol-related liver disease (ARLD) is a global health issue causing significant morbidity and mortality, due to lack of suitable therapeutic options. ARLD induces a spectrum of biochemical and cellular alterations, including chronic oxidative stress, mitochondrial dysfunction, and cell death, resulting in hepatic injury. Natural antioxidant compounds such as curcumin have generated interest in ARLD due to their ability to scavenge reactive oxygen species (ROS), however, therapy using these compounds is limited due to poor bioavailability and stability. Therefore, the aim of this study was to assess the antioxidant potential of free antioxidants and curcumin entrapped formulations against oxidative damage in an ARLD cell model. HepG2 (VL-17A) cells were treated with varying concentrations of alcohol (from 200 to 350 mM) and parameters of oxidative stress and mitochondrial function were assessed over 72 h. Data indicated 350 mM of ethanol led to a significant decrease in cell viability at 72 h, and a significant increase in ROS at 30 min. A substantial number of cells were in late apoptosis at 72 h, and a reduction in the mitochondrial membrane potential was also found. Pre-treatment with curcumin nanoformulations increased viability, as well as, reducing ROS at 2 h, 48 h and 72 h. In summary, antioxidants and entrapped nanoformulations of curcumin were able to ameliorate reduced cell viability and increased ROS caused by ethanol treatment. This demonstrates their potential at mitigating oxidative damage and warrants further investigation to evaluate their efficacy for ARLD therapy.

## Introduction

Alcoholic related liver disease (ARLD) is a progressive chronic disease which develops due to excessive and prolonged alcohol exposure resulting in steatosis, alcoholic hepatitis, fibrosis/cirrhosis and in some cases, hepatocellular carcinoma^1^. Liver disease is a significant global health issue, responsible for around two million deaths per year and 4% of all deaths worldwide^2^. The World Health Organization’s (WHO) Global Status Report on Alcohol and Health estimates 2.3 billion people currently consume alcohol, with an average intake daily of 32.8 g of alcohol per person^3^. Multiple factors have been identified as inducers of liver inflammation in response to alcohol. These factors include mitochondrial dysfunction, gut dysbiosis, alcohol metabolites, necrotic cell products such as damage associated molecular patterns (DAMPs), apoptosis and mitophagy^4^. These factors are highly interactive with common downstream intermediates, in particular, reactive oxygen species (ROS), which results in a state of oxidative stress^5^. Furthermore, the highly reactive by-product of alcohol metabolism, acetaldehyde, can form protein and DNA adducts, generate ROS, and stimulate an immune response^6–9^, thus promoting inflammation. Disruption to the mitochondrial electron transport chain (ETC) and oxidative phosphorylation is also a common feature in ARLD, leading to necroptosis^10,11^. However, the exact mechanisms of inflammation, mitochondrial dysfunction and pro-inflammatory cytokines production requires further research^12,13^.

Abstaining from alcohol is considered the primary therapeutic approach for individuals attending rehabilitation centres^1,14^, where abstinence from alcohol has demonstrated a reduction in portal pressure, a decreased likelihood of advancing to cirrhosis, and an improvement in survival rates^14,15^. Unfortunately, abstaining from alcohol has high relapse rates, ranging from 67 to 81% among alcoholics^16^. Research has also shown that 60% of patients with alcohol use disorder will relapse within 6 months. Furthermore, limited therapeutic options exist for the treatment of severe alcoholic hepatitis which has a mortality rate of up to 45%^17^ and limited studies exist which indicate improvement in survival rates amongst alcoholic hepatitis patients^18,19^. Clinical trials for ARLD have not been successful, including the highly anticipated Steroids or Pentoxifylline for Alcoholic Hepatitis (STOPAH) trial^20,21^. Other clinical trials for ARLD which have not been successful; include IFN-γ, angiotensin II antagonists and IL-10^22^. Therefore, this highlights the need for alternative treatment approaches capable in mitigating oxidative stress and liver injury, a critical step in disease pathology.

Glutathione (GSH) is the main cellular antioxidant synthesised by S-adenosyl methionine (SAM) and can control cellular redox and protect against oxidative stress, neutralising ROS, however, during ARLD development, GSH levels are depleted. Antioxidant therapy has been considered useful for ARLD treatment as many antioxidant compounds can mediate ROS including vitamins E and C, *N*-acetylcysteine (NAC), SAM, and betaine^9,23^. Curcumin has also been shown to have antioxidant properties and can reduce oxidative stress via ROS reduction in animal models^24^. Although curcumin possesses beneficial antioxidant properties it has low bioavailability and stability issues, therefore, the ability to achieve targeted therapeutic doses becomes limited. Nanoformulations may mitigate some difficulties faced with traditional drugs due to their ability to deliver drugs to specific cell types based on surface receptor binding^22^, as well as controlled drug release, specific cell penetration, improved pharmacokinetics, and reduced side effects^25^. Novel nanocarrier delivery systems entrapping curcumin have previously been shown to protect against oxidative stress in a Parkinson’s disease model^26^.

The combination of entrapped antioxidants for the treatment of ARLD may provide a promising approach compared to traditional treatments in the ability to reduce oxidative stress. Therefore, the aim of this study was to utilise novel nanocarrier systems entrapping potent antioxidants such as curcumin to assess their ability to protect against ethanol induced damage in a liver cell model.

## Materials and methods

### Cell culture

HepG2 (VL-17A) cells (kindly provided by Dr Clemens, University of Nebraska) which overexpress both ADH and CYP2E1 were cultured in Dulbecco’s Modified Eagles Medium (Lonza Ltd, UK) supplemented with 10% foetal calf serum (FCS) (Gibco, UK)^27–31^, 10 U/mL penicillin/streptomycin, 2 mM l-glutamate and 50 μg/mL gentamycin sulphate (Lonza, UK), which has been previously characterised when studying fatty liver and alcohol toxicity mechanisms^31–34^. When cells reached 70% confluency, they were passaged using a 0.5× trypsin solution in phosphate buffered saline (PBS) and viability of cells was assayed using trypan blue.

### Cell treatments

Cells were seeded according to the appropriate protocol (as described below) and treated with varying concentrations of ethanol (100, 200 mM, 300 mM, 350 mM) in low glucose (1.0 g/L) DMEM supplemented with 1% (v/v) foetal calf serum (FCS), 1% penicillin/streptomycin (100 U/mL) and 1% l-Glutamine (2 mM).

These alcohol concentrations were chosen, as we have previously shown that in HepG2 cells that overexpress ADH, 100 mM alcohol treatment led to a moderate decrease in cell viability and mitochondrial function^34^. As a consequence, to induce more cellular injury that reflects severe alcoholic hepatitis, higher alcohol concentrations were characterized up to 350 mM. The concentration is in alignment with other in vitro studies using HepG2 cells from 171 to 750 mM^27,35–37^; 100–800 mM in a L-02 hepatocyte line^38^, 200 mM in a mouse liver cell line^39^, 300 mM in neuronal cells^40^. For cell culture, the original methodology as stated by Clemens et al., was followed where flasks were tightly sealed or cell culture plates were sealed to prevent ethanol evaporation^29,41^. To determine whether nanocarrier systems could protect against oxidative damage VL17-A cells were also treated with antioxidant compounds and the above nanoformulations (produced in collaboration with UCL) at differing concentrations and time points as either a pre-treatment prior to ethanol or, added as co-treatment with ethanol. The varying treatments were studied using parameters of liver toxicity over 30 min to 72 h. The effects of cell treatment were assessed by the methods below.

### Cell viability

Cells were seeded at 1 × 10^4^ cell/well and incubated overnight to allow attachment. Following treatment, 5 mg/mL 3-(4,5-Dimethylthiazol-2-yl)-2,5-diphenyltetrazolium bromide (MTT) was added to each well and incubated at 37 °C for 2 h^31^. After incubation, the reagent was removed, and cells were incubated with 100 µL/well DMSO for 15 min at room temperature. The plates were then measured at 550 nm.

### Reactive oxygen species assay

The level of ROS production was assayed using a method adapted from Baldini et al.^42^. Cells were seeded at 1 × 10^4^ cell/well and incubated overnight to allow attachment. Following treatment, the cells were washed twice with PBS and incubated with 200 μL of 200 µM 2, 7-dichlorofluoresceindiacetate (DCF-DA) (Sigma, UK) and incubated at 37 °C for 30 min in the dark. Following incubation, the substrate solution was removed, and cells were washed in PBS. 200 μL of PBS was then added to each well and fluorescence was measured at excitation 485 nm and emission 535 nm.

### Measurement of apoptosis

To determine quantities of apoptotic cells after treatment, the Annexin VFITC/propidium iodide (PI) staining kit (BioLegend, UK) was used to stain cells and measured by flow cytometry. 3 × 10^5^ cells were seeded in 12-well plates and left overnight to attach. Cells were treated as described above over 72 h. After treatment, supernatant of cells was kept, and cells were then washed with PBS prior to detachment from plates with 200 µL trypsin (incubated at 37 °C for 5 min). After detachment, 800 µL of 10% FCS DMEM was then added to neutralise the trypsin and the cell/supernatant mixture was then centrifuged at 400 g for 5 min. Cell pellets were resuspended in 500 µL of 1X Annexin V Binding Buffer and stained with 5 µL of Annexin V-FITC and 5 µL PI and analysed on the BD LSRFortessa™ (Ex = 488 nm, Em = 530 nm) using the FACSDiva software.

### Measurement of mitochondrial respiration

HepG2 cells were plated in 24-well Seahorse MitoStress Assay cell plates at a density of 2 × 10^4^ cells per well. Cells were then treated with the previously described conditions of alcohol. After the defined treatment length, cells were washed with Seahorse Assay Medium (pH 7.4) containing 25 mM glucose and 1mM sodium pyruvate^31^. MitoStress drugs oligomycin (4 µM), FCCP (4 µM), and antimycin/Rotenone mixture (2.5 µM) were added, and the oxygen consumption rate (OCR) was measured using the Seahorse XFE Flux Analyzer under basal conditions. OCR values were normalised to total protein content which was measured using the Bradford Assay^43^ (Bio-Rad Laboratories, UK).

### Measurement of genome damage

The cytokinesis-block micronucleus cytome assay was performed as described by Michael Fenech^44^ to analyse measures of genome damage and chromosomal instability. 1 × 10^5^ cells were seeded in T25 flasks, and the following day treated as described above. Cytochalasin B was added to media for 24 h prior to their fixation. Following this, cells were then detached from flasks using trypsin and centrifuged at 400 g. After centrifugation, the supernatant was discarded and then replaced with 7 mL prewarmed 0.075 M potassium chloride and incubated at 37 °C for 7 min. Cells were then centrifuged and potassium chloride was removed and replaced with 5 mL fixative (methanol:acetic acid (3:1)). For analysis, cells were centrifuged and resuspended in 500 μL cold fixative and dropped onto microscope slides then stained with DAPI and 5% Giemsa. The number of micronuclei (MNi), nucleoplasmic bridges (NPBs), and nuclear buds (NBUDs) were scored from 100 binucleated cells.

### Preparation of antioxidant nanoformulations

The concentrations of curcumin (5 and 10 μM) used in this study were optimised based on previous studies from our research group^26,45^ and existing literature^46,47^. Concentrations of 5 µM and 10 µM have been found to serve as the optimal concentration in preclinical studies for assessing absorption efficiency and assessing the potential of bioenhancement strategies, such as nanoformulations and liposomal encapsulations. Determining the pharmacokinetic profile at 5 µM and 10 µM concentrations helps define the therapeutic window of curcumin, which can potentially identify the minimum effective concentration needed to produce a pharmacological effect. These optimised concentrations were thus chosen to ensure measurable and reproducible effects while minimising cytotoxicity.

All nanoformulations were prepared using a modified thin-film hydration method^48,49^. Curcumin entrapment in nanoformulations were prepared with 100% DSPE-PEG^50,51^. A rotary evaporator (Buchi Rotavapor^®^ R-100, Flawil, Switzerland) was then used to evaporate the methanol (200 rpm and 80 °C), under vacuum^45,52–54^. Once a thin film was achieved, it was then hydrated with 10 mL of Milli-Q water and mixed at 80 °C then sonicated using a VWR Ultrasonic cleaner bath USC300T (VWR International Limited, UK) for a further 5 min^45,52–54^. The solution was then filtered through a sterile 0.45 μm filter to remove any unloaded antioxidants^45,52–54^.

### Size and surface charge of nanoformulations

The size and surface charge of nanoformulations were measured by dynamic light scattering (DLS) as Z_Ave_ hydrodynamic diameter, polydispersity index (PDI) and zeta potential (§), using the Zetasizer Nano ZS (Malvern Instruments, UK)^45,52–54^. 1 mL of the nanoformulated sample was pipetted into the zeta potential DTS1070 folded capillary cell (Malvern Panalytical, UK). Zeta potential was calculated via electrophoretic mobility using Malvern data analysis software following the Helmholtz-Smoluchowski equation^45,52–54^.

### Determination of drug loading and entrapment efficiency

Drug loading and entrapment efficiency of nanoformulations were measured by UV–Visible spectrophotometer (Cary Series UV–Vis spectrophotometer, Agilent Technologies, USA) using free drug calibration curves^45,52–54^. Curcumin was measured at 424 nm. The percentage of drug loading and entrapment efficiency were calculated using the following equations^54^:

$$Drug\, loading \left(\%\right)=\frac{total\,weight\, of\, entrapped\, drug}{total\, weight\, of\, all\, raw\, materials}\times 100$$

$$Encapsulation\, efficiency \left(\%\right)=\frac{(determined\, mass\, of\, drug\, entrapped\, within\, nanocarriers)}{actual\, mass\, of\, drug}\times 100$$

### Statistical analysis

Results were analysed using either one or two-way ANOVA. Statistical analysis was followed by Tukey’s multiple comparisons post hoc test. Data is expressed as mean + standard error of the mean (SEM) and P values ≤ 0.05 were considered statistically significant.

## Results

**Fig. 1:**
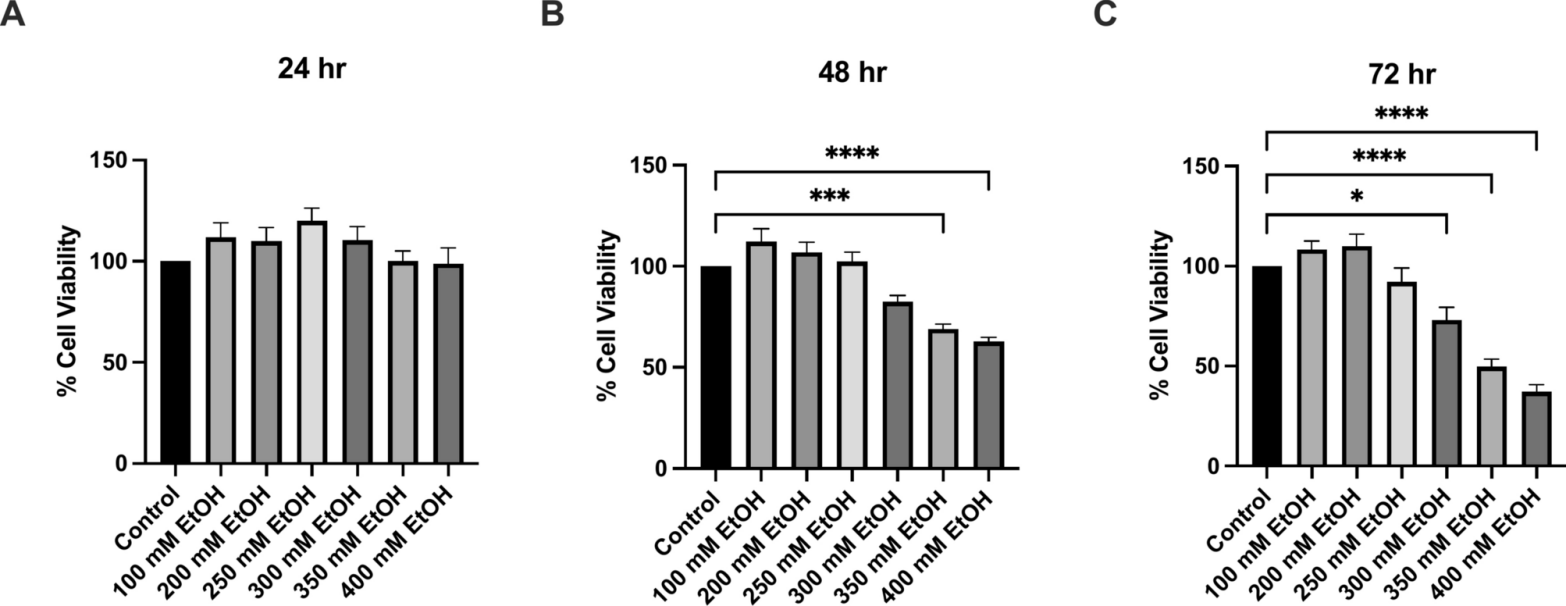
The effect of ethanol exposure on cell viability at (**A**) 24 h, (**B**) 48 h and (**C**) 72 h. Data is presented as percentage from the control. Results presented as mean of replicates ± SEM (n = 9) *P ≤ 0.05, ***P ≤ 0.001, ****P ≤ 0.0001.

HepG2 (VL-17A) cells were exposed to varying concentrations of ethanol (100–400 mM) and cell viability was assessed. At 48 h, an 18% decrease in cell viability was observed after 300 mM ethanol exposure, 350 mM ethanol led to a 31% decrease (p = 0.0005) and 400 mM also led to a 37% decrease (p < 0.0001) (Fig. 1). Ethanol toxicity was most apparent after 72 h whereby 300 mM alcohol led to a 27% decrease (p = 0.0438), 350 mM led to a 50% decrease (p < 0.0001) and 400 mM a 63% decrease (p < 0.0001) (Fig. 1C).

**Fig. 2:**
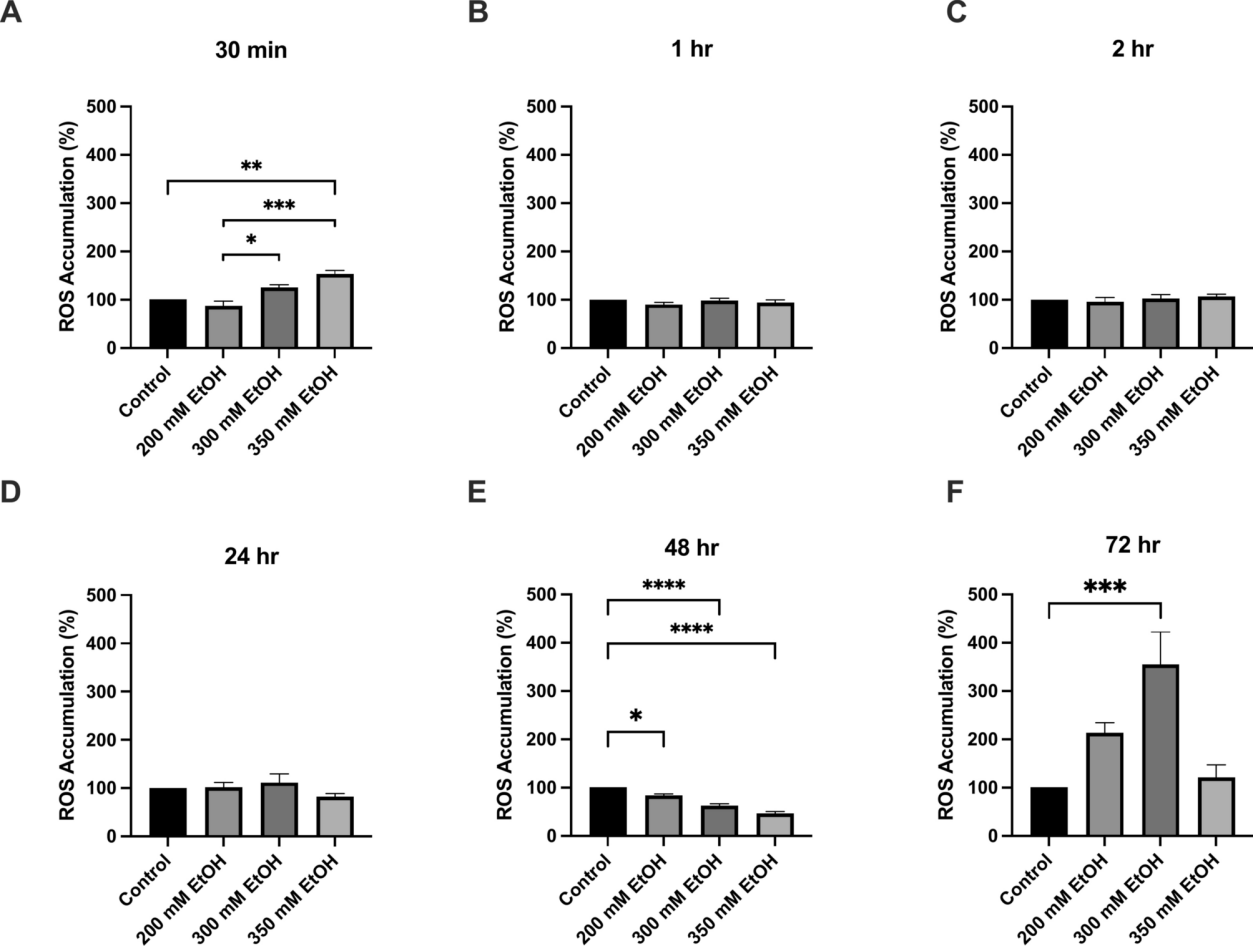
The effect of alcohol exposure on ROS accumulation at (**A**) 30 min, (**B**) 1 h, (**C**) 2 h, (**D**) 24 h, (**E**) 48 h and (**F**) 72 h. Cells were seeded in 96-well plates and treated with 200 mM, 300 mM, and 350 mM ethanol. Data is presented as percentage from the control. Results are expressed as mean of replicates ± SEM (n = 3–6). *P ≤ 0.05, **P ≤ 0.01, ***P ≤ 0.001, ****P ≤ 0.0001.

**Fig. 3:**
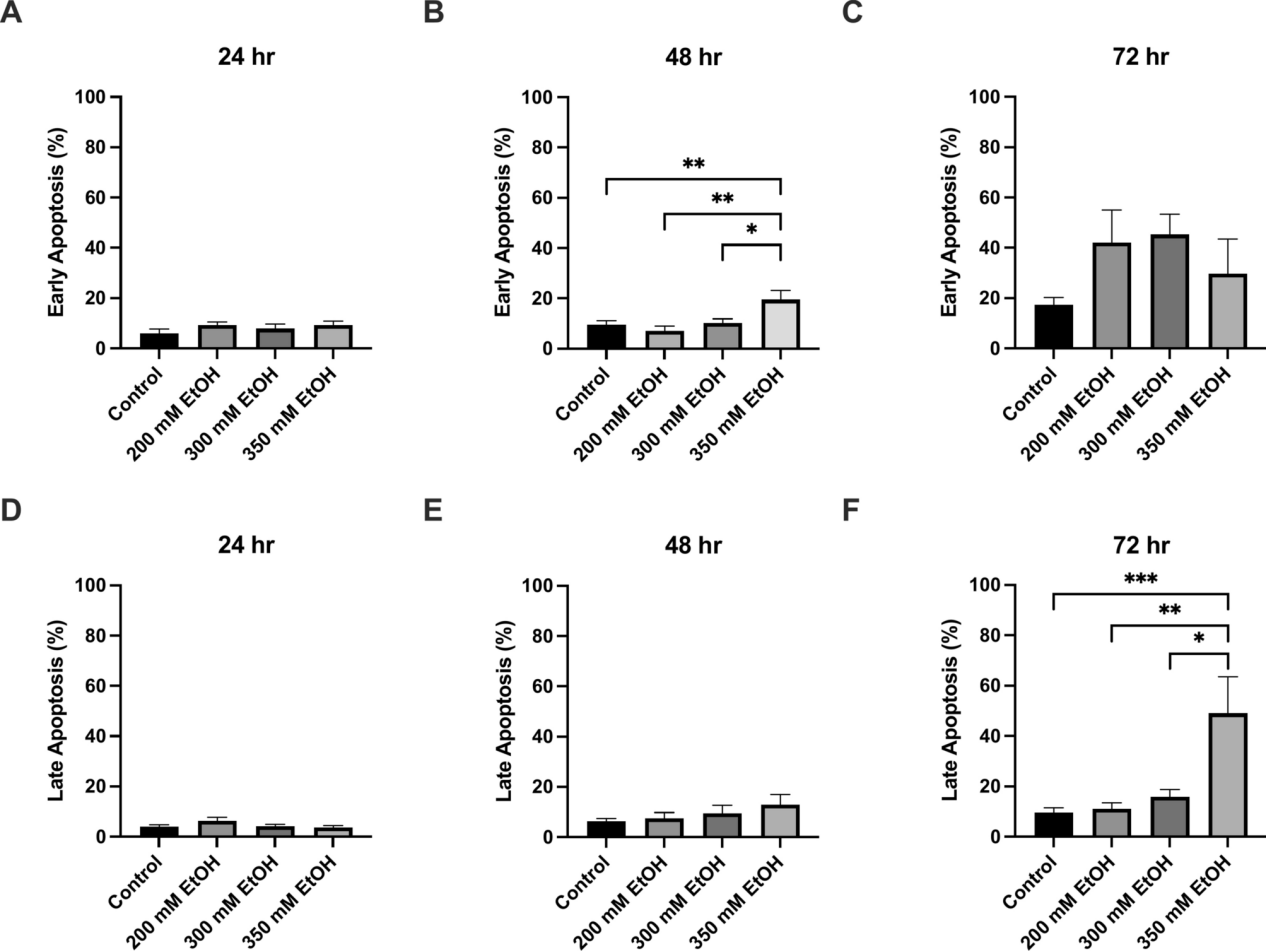
The effect of ethanol exposure on apoptosis (**A**) 24 h early apoptosis (**B**) 48 h early apoptosis, (**C**) 72 h early apoptosis, (**D**) 24 h late apoptosis (**E**) 48 h late apoptosis, (**F**) 72 h late apoptosis. Data is presented as percentage of positive cells. Results presented as mean of replicates ± SEM (n = 3–10). *P ≤ 0.05, **P ≤ 0.01, ***P ≤ 0.001.

**Fig. 4:**
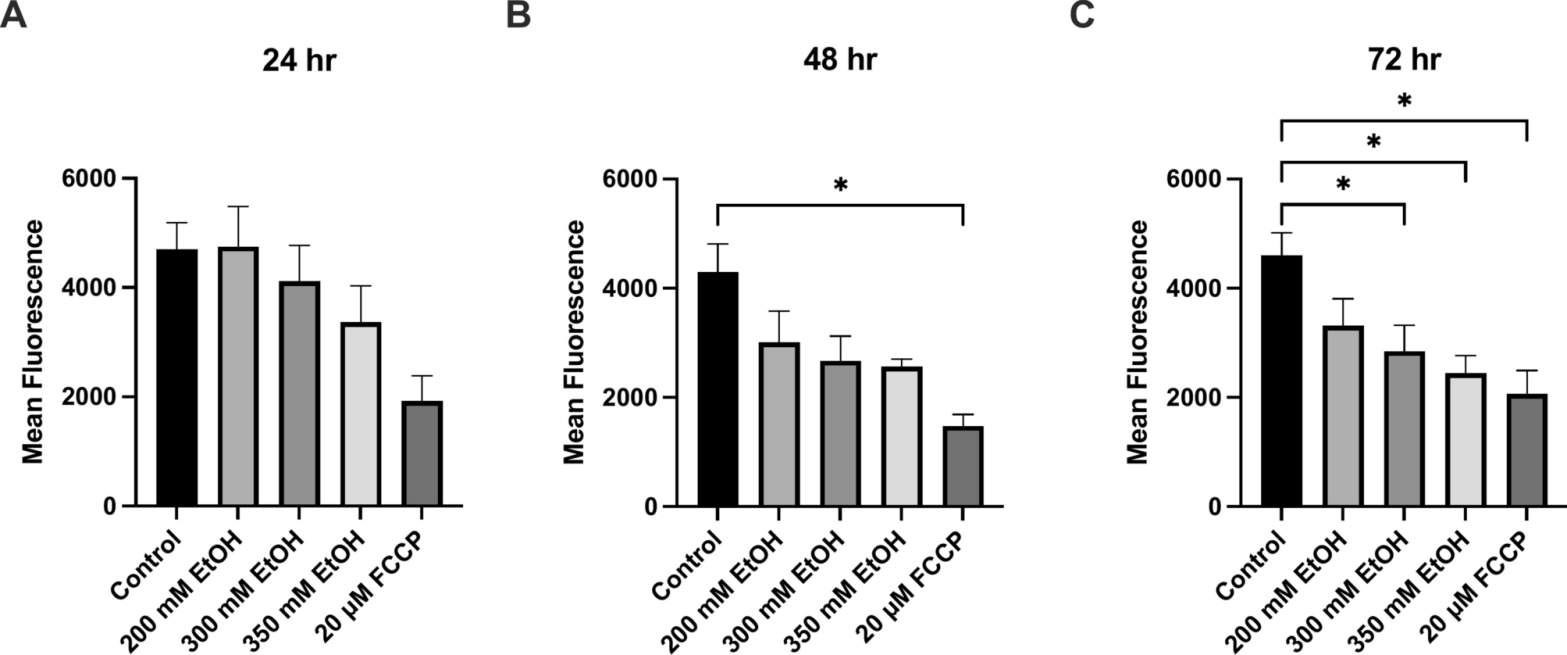
The effect of ethanol on mitochondrial membrane potential at (**A**) 24 h, (**B**) 48 h and (**C**) 72 h. FCCP (20 μM) was used as a positive control. Data is presented as mean fluorescence values. Results presented as mean of replicates ± SEM (n = 3). *P ≤ 0.05.

At 30 min, a 25% increase in ROS was seen after treatment with 300 mM and a 53% increase (p = 0.0027) after treatment with 350 mM ethanol (Fig. 2). Similarly, at 72 h, 200 mM ethanol exposure increased ROS by 42%, and 300mM increased ROS by 96% (p = 0.0005) (Fig. 2F). At 48 h, the percentage of cells in early apoptosis also increased significantly with 20% (p = 0.0313) of cells treated with 350 mM ethanol in early apoptosis (Fig. 3). Overall, at 72 h, both the percentage of early and late apoptosis was highest, and 350 mM ethanol caused 44% of cells to undergo late apoptosis (p = 0.0153) (Fig. 3). A dose dependent decrease in mitochondrial membrane potential was also observed across all time points (Fig. 4). Mitochondria play a crucial role in ROS formation and cellular energy metabolism and recent research indicates that alcohol consumption can induce alterations in both the structure and function of mitochondria. The assessment of mitochondrial respiratory function was conducted by measuring the OCR (Supplementary Fig. S1). At 24 h, measurements of oxygen consumption were also reduced (Supplementary Fig. S2). Preliminary findings indicate that ethanol treatment leads to an increase in measures of chromosomal instability, with significant increases in NBUD formation (Supplementary Fig. S4).

**Fig. 5:**
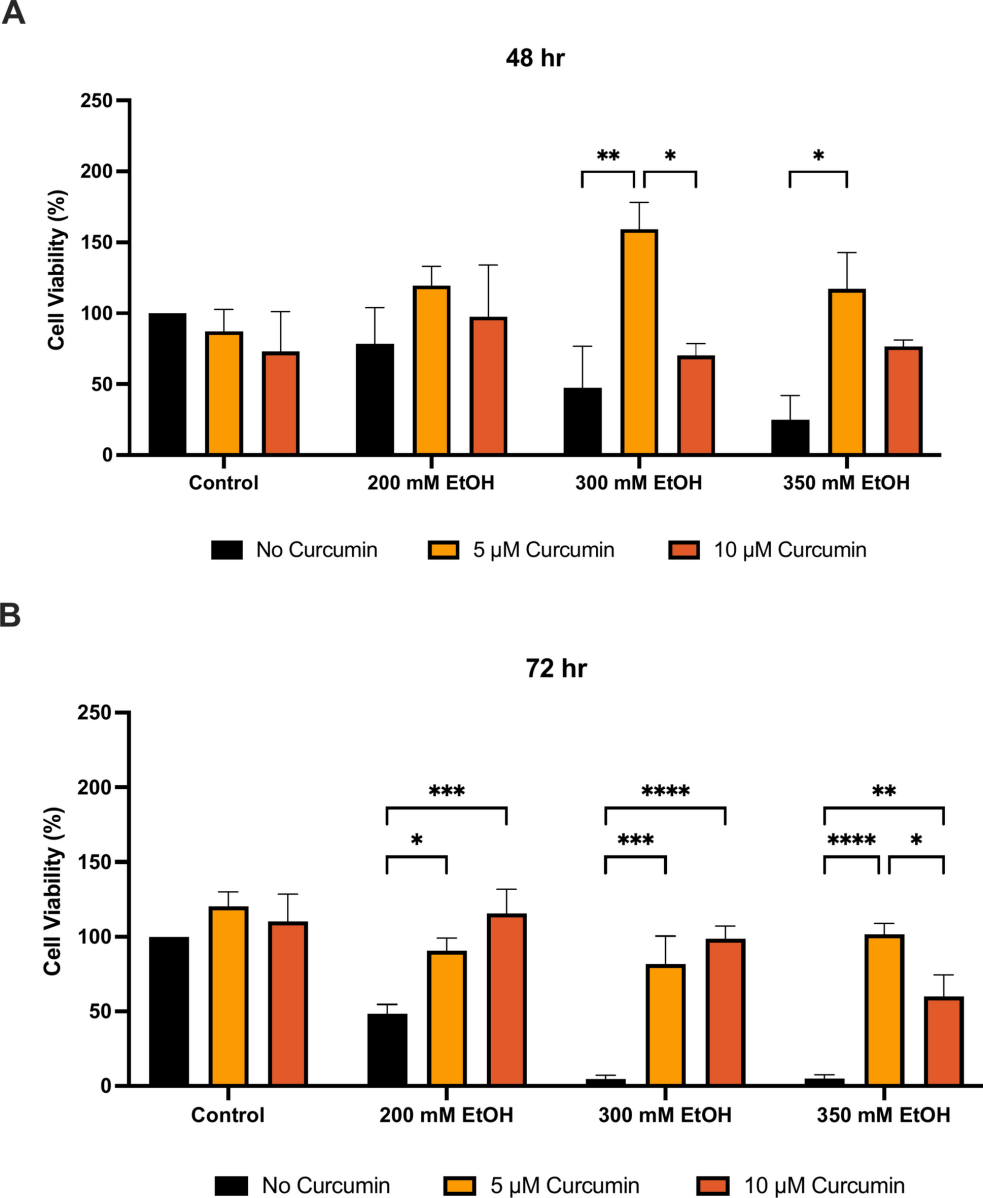
The effect of curcumin free drug co-treatment with and ethanol on cell viability at (**A**) 48 h and (**B**) 72 h. Cells were seeded in 96-well plates and co-treated with ethanol and curcumin. Viability was determined by MTT assay. Data is presented as percentage from the control. Results presented as mean + SEM (n = 3) *P ≤ 0.05, **P ≤ 0.01, ***P ≤ 0.001, ****P ≤ 0.0001.

Free drug curcumin was tested in its ability to prevent cellular injury due to alcohol. At 48h, 200 mM ethanol led to a 22% reduction in viability, 300 mM led to a 53% reduction and 350 mM led to a 75% reduction in viability and at 72 h mM ethanol led to a 52% reduction in viability and 300 mM and 350 mM both led to a 95% reduction in viability. 5 μM and 10 μM curcumin were assessed, and this data shows that when curcumin was included with ethanol it provided protection against ethanol and induced damage to HepG2 cells. At 48 h, 5 μM free drug was able to increase viability by 237% with 300 mM ethanol (p = 0.0030) and 369% with 350 mM ethanol (p = 0.0141), when compared to the ethanol treated only controls (Fig. 5). At 72 h, 5 μM free drug was able to increase viability by 88% with 200 mM ethanol (p = 0.0335), 1650% with 300 mM ethanol (p = 0.0002), and 1933% with 350 mM ethanol (p < 0.0001), when compared to the ethanol treated only controls (Fig. 5). 10 μM free drug was able to increase viability by 139% with 200 mM ethanol (p = 0.0008), 2014% with 300 mM ethanol (p < 0.0001), and 1100% with 350 mM ethanol (p = 0.0052), when compared to the ethanol treated only controls (Fig. 5).

Novel nanocarrier delivery systems for the antioxidant curcumin were used to assess their ability in the protection against ethanol induced changes in VL-17A cells. Formulations of curcumin DSPE-PEG nanoformulation exhibited notably high encapsulation efficiency, ranging from 74 to 78% (Table 1). The average particle sizes of the nanoformulation, as measured by diameter, were consistently below 10 nm. Specifically, the mean diameter for DSPE-PEG nanocarrier was 8.40 nm and the polydispersity index (PDI) values, reflecting the heterogeneity of nanocarrier solutions, were low (< 0.5). The evaluation of Zeta Potential (mV) was employed to assess the surface charge of the nanoformulations. Nanocarriers exhibited comparable low negative surface charges, measuring -15.20 mV.

**Table 1:** Nanoformulation characteristics. Hydrodynamic diameter (d), polydispersity index (PDI), surface charge, drug loading (DL) and encapsulation efficiency (EE) of curcumin DSPE-PEG nanoformulations prepared at 80 °C (mean ± SEM n = 3).

Active ingredient (10 mg)	Polymer (100 mg)	d (nm)	PDI	Zeta potential (mV)	DL (%)	EE (%)
Curcumin	100% DSPE-PEG	8.40 ± 0.67	0.43 ± 0.03	− 15.20 ± 0.95	7.11 ± 0.00	78.25 ± 0.02

**Fig. 6:**
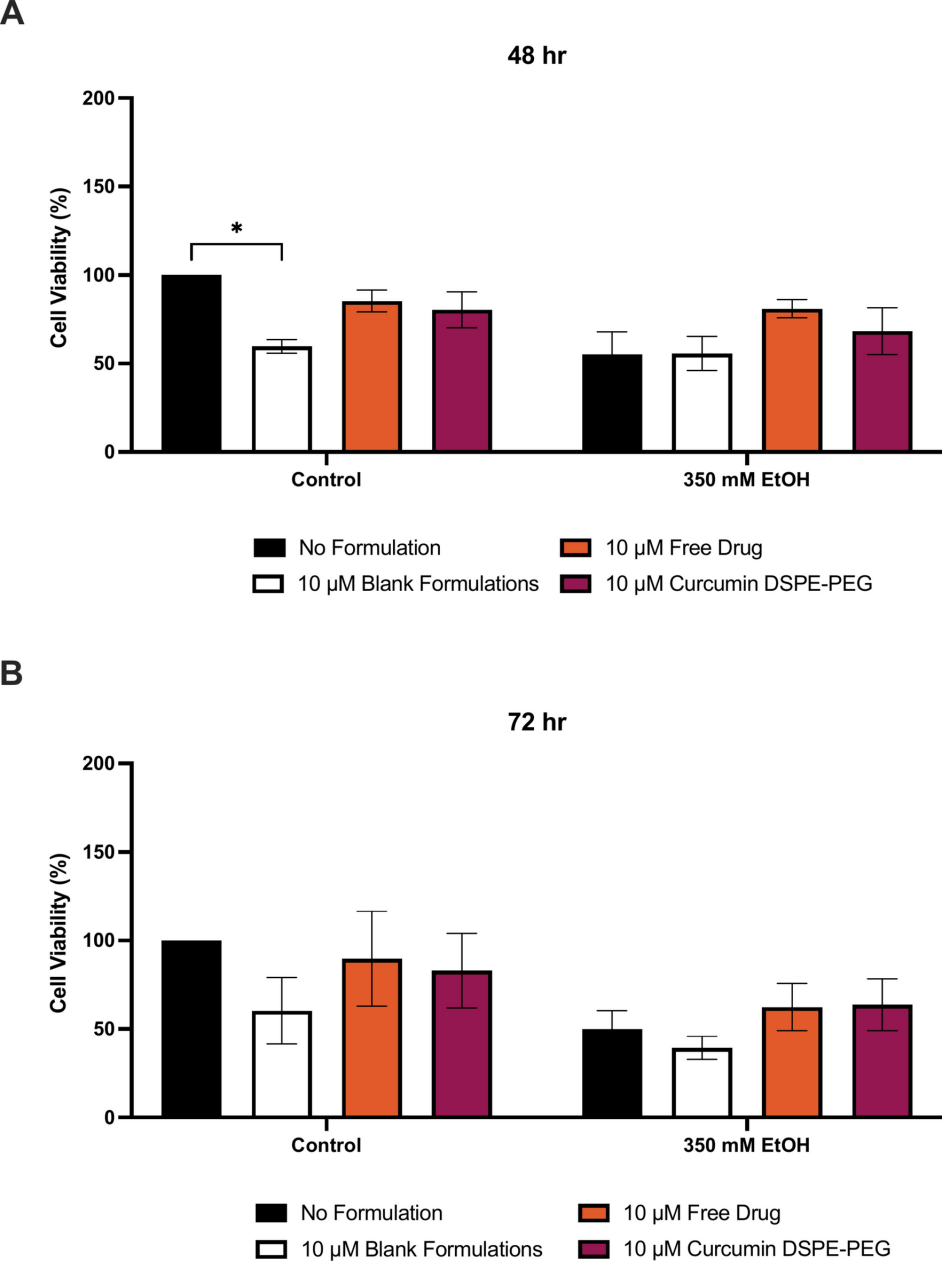
3-h pre-treatment with nanoformulated curcumin on loss of cell viability at (**A**) 48 h and (**B**) 72 h. Cells were seeded in 96-well plates and treated with 350 mM ethanol after 3 h pre-treatment of formulations. Viability of cells was determined by an MTT assay and measured at 48 h and 72 h. Data is presented as percentage from the control. Results presented as mean + SEM (n = 3) *P ≤ 0.05.

Protection with curcumin formulated in DSPE-PEG carrier was assessed using MTT assay (Fig. 6). At 48 h, 350 mM ethanol caused a reduction in cell viability by 45%. Pre-treatment with 10 μM curcumin DSPE-PEG increased viability by 24% when compared to 350 mM ethanol treatment alone (Fig. 6). At 72 h, 350 mM ethanol caused a reduction in cell viability by 50%. Pre-treatment with 10 μM curcumin DSPE-PEG increased viability by 27% when compared to 350 mM ethanol treatment alone (Fig. 6).

**Fig. 7:**
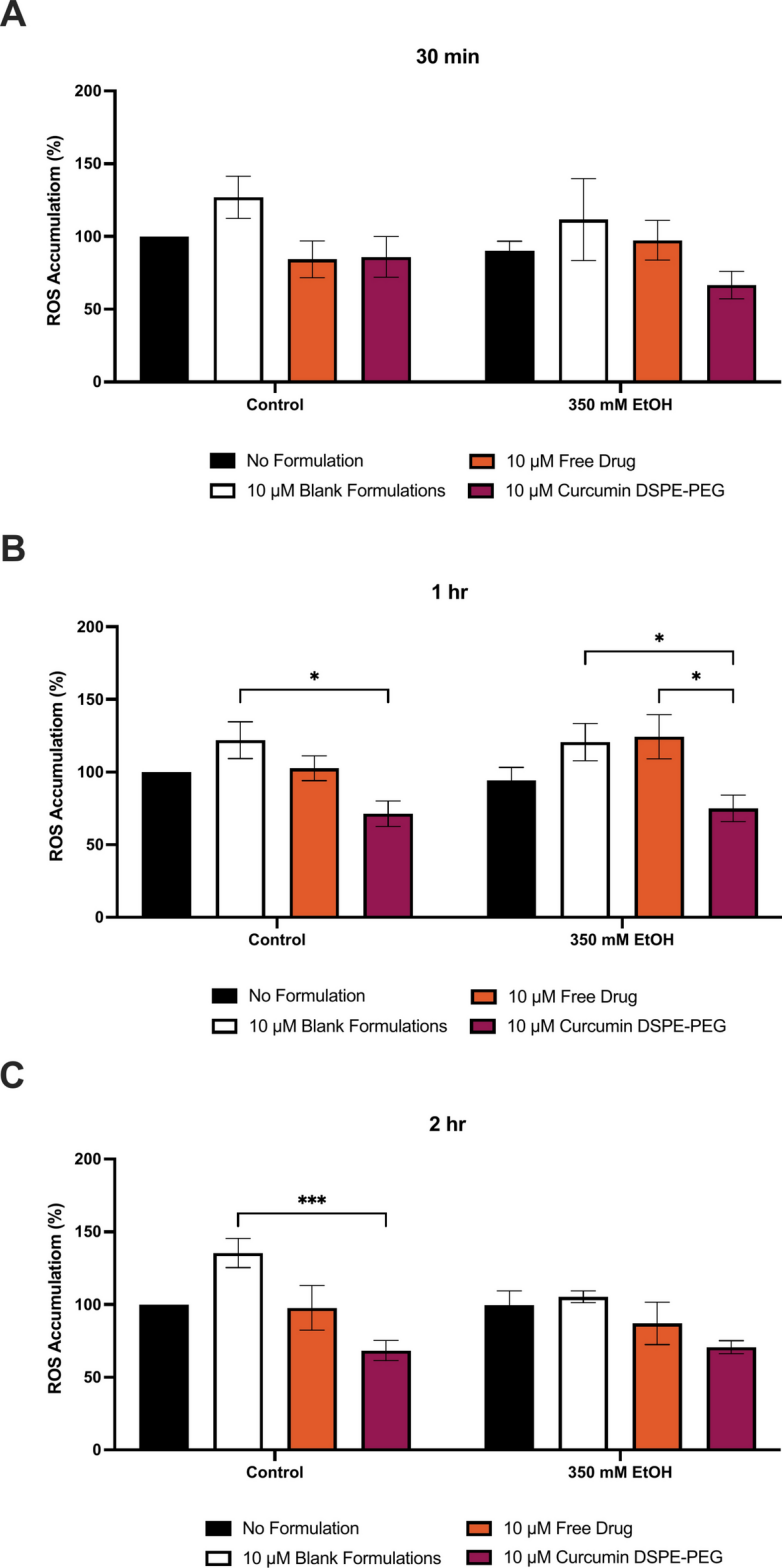
3 h pre-treatment of nanoformulated curcumin on ethanol induced ROS production at (**A**) 30 min, (**B**) 1 h and (**C**) 2 h. Cells were seeded in 96-well plates and treated with 350 mM ethanol after  3 h pre-treatment of formulations. ROS production was determined by the DCFDA assay. Data is presented as percentage from the control. Results presented as mean + SEM (n = 3) *P ≤ 0.05, ***P ≤ 0.001.

**Fig. 8:**
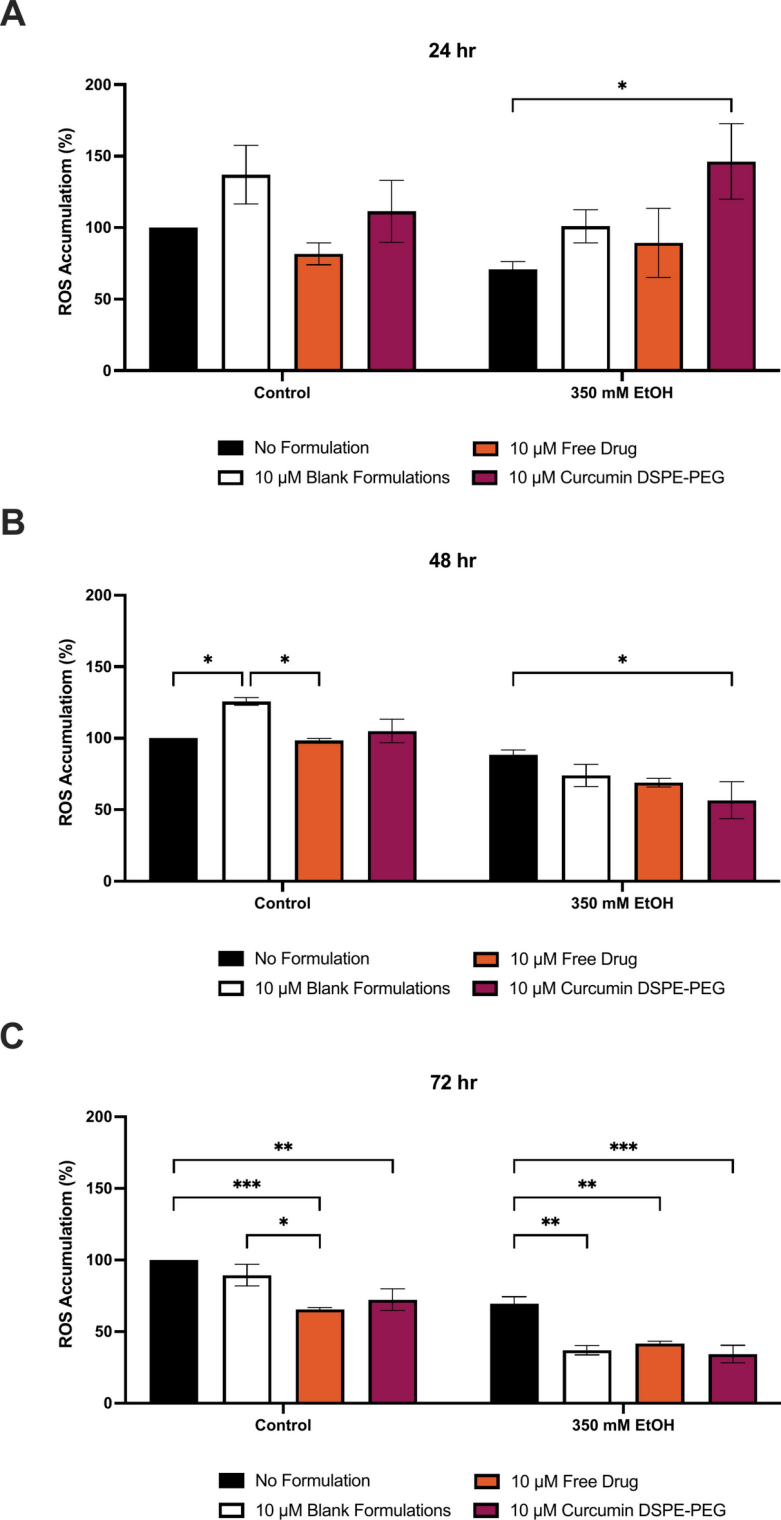
3 h pre-treatment of nanoformulated curcumin on ethanol induced ROS production at (**A**) 24 h, (**B**) 48 h and (**C**) 72 h. Cells were seeded in 96-well plates and treated with 350 mM ethanol after 3 h pre-treatment of formulations. ROS production was determined by the DCFDA assay. Data is presented as percentage from the control. Results presented as mean + SEM (n = 3) *P ≤ 0.05, **P ≤ 0.01, ***P ≤ 0.001.

Curcumin DSPE-PEG nanoformulations were shown to protect VL-17A cells against ROS production (Figs. 7, 8). At 30 min, pre-treatment with 10 μM curcumin DSPE-PEG reduced ROS by 26% and at 1 h, 10 μM curcumin DSPE-PEG a 20% reduction was observed, compared to corresponding ethanol only treatment. At 2 h, pre-treatment with 10 μM curcumin DSPE-PEG reduced ROS by 29%. At 48 h, pre-treatment with 10 μM curcumin DSPE-PEG reduced ROS by 36% (p = 0.0226) and at 72 h pre-treatment with 10 μM curcumin DSPE-PEG significantly reduced ROS by 51% (p = 0.0013).

## Discussion

ARLD is a significant global health issue, however, the precise mechanisms for liver injury are still incompletely understood. This in part explains the limited treatment options available, particularly for acute alcoholic hepatitis which has a 60-day mortality rate of 20–30%^55^, which increases to 40% at 180 days after admission^17^. We have previously shown that 100 mM ethanol in HepG2 cells causes a moderate decrease in cell viability and mitochondrial function (loss in membrane potential and lower ATP levels)^34^. This suggested that higher ethanol concentrations were required to induce more pronounced cellular injury, and thus we could evaluate the antioxidant properties of nanoformulations under more damaging conditions that reflects severe alcoholic hepatitis. This is similar to other models of ARLD, where liver slices were exposed to 250 mM and 500 mM ethanol^56^.

In this study, HepG2 (VL-17A) cells were used as a model to investigate alcohol-induced injury in liver cells in relation to oxidative stress, apoptosis, mitochondrial dysfunction, and genomic damage. Ethanol treatment led to significant cell toxicity, indicating a correlation between alcohol dosage and reduced cell viability over time. This study also found increased levels of ROS and apoptosis as well as decreases in mitochondrial membrane potential, suggesting ethanol treatment causes oxidative stress as well as disrupts mitochondrial cellular energy production, which in turn, causes activation of apoptosis. Results also show ethanol treatment leads to chromosomal instability and genome damage.

Mitochondrial dysfunction occurs due to the increase in ROS production and oxidative stress. During alcohol metabolism, excess NADH is produced which alters the cellular redox status and drives complex I of the ETC, causing electron leakage from complexes I and III, and the generation of superoxide anion radical and hydrogen peroxide^9,57–60^. *In-vivo*, alcohol has also been shown to increases gut dysbiosis, causing LPS translocation and production of pro-inflammatory cytokines such as TNF-α, and IL-8, which in turn, can induce apoptosis^9,61^. Apoptosis can also be induced due to protein adduct formation which elicits an immunological response and production of IFN-γ and TNF-α^61^. Induction of apoptosis may also occur due to hypoxia and reduced levels of mitochondrial GSH^62^. Therefore, increases observed in apoptosis may be due to the reduction in mitochondrial membrane potential, dysfunctional cellular redox status and reduced levels of antioxidants.

In the present study an increase in late apoptosis was observed. The late phase of apoptosis is marked by DNA fragmentation, as well as morphological and phenotypic changes. Previous research has shown that ethanol treatment causes increases in DNA fragmentation^63^ as well as DNA damage and chromosomal and genomic instability^64,65^, which has been confirmed by the present study regarding ethanol and genomic instability.

It is well known that excessive alcohol causes oxidative stress and DNA damage. When DNA damage occurs, chromosomal and genomic instability can increase which leaves cells susceptible to oxidative stress, inflammation and cancer^64^. Data in this study shows that treatment with various concentrations of ethanol causes increased numbers of micronuclei and nuclear buds, suggesting excessive alcohol treatment may induce cancer initiation via DNA damage^64^. Although the mechanisms are not well known, acetaldehyde, the toxic by-product, can bind to DNA producing point mutations^66^. In addition, acetaldehyde disrupts mitochondrial GSH uptake, electron transport chain activity and directly impairs mitochondrial respiration^67–69^.

Whilst most of the toxicity arose at the latter time points, it is important to demonstrate in the current study the transitional dose and time dependent effects of alcohol treatment from 24 to 72 h. It is likely the initial increase in ROS production, arises due to immediate alcohol metabolism, whereas there is a lag time in cellular injury, with minimal changes in viability occuring at 24 h, and viability decreasing at latter time points 48–72 h. We have previously reported that *in-vivo,* alcohol toxicity occurs over a 24–72 h period in the hepatic cytosol and mitochondria, with specific changes in oxidative stress. Furthermore, acetaldehyde causes further detrimental effects over the 72 h period^68^, highlighting the differential time dependent effects of alcohol and acetaldehyde.

Due to the significant contribution of oxidative stress to the inflammatory process in ARLD, antioxidant therapy has been considered as a treatment approach. There is substantial evidence supporting the protective effects of curcumin in many disorders including neurological disease, liver disease, cancer, diabetes and cardiovascular diseases^70–73^. However, the use of these antioxidants for potential therapy has been hindered by their low bioavailability, low stability and low concentration at the target site. Nanomedicine is an emerging technology which offers promising solutions to these problems as they can preserve the potency of the entrapped compound while improving targeted delivery and crossing biological membranes.

Novel nanocarrier delivery systems have become of recent interest as they aim to enhance stability, bioavailability, and delivery of the entrapped compounds to specific cells. Formulations have also been developed to increase solubility^74^. The aim of this study was to use DSPE-PEG nanocarrier systems to entrap potent antioxidant compounds such as curcumin to assess their ability in protecting against oxidative stress in ARLD. Curcumin has previously been shown to exhibit its antioxidant, anti-inflammatory and anticarcinogenic property on the liver. Previous research has shown that hydrogen peroxide treatment on HepG2, inducing oxidative stress, can be mitigated by the effects of curcumin which was shown to reduce ROS and malondialdehyde (MDA) formation (a by-product of lipid peroxidation), as well as increase antioxidant enzyme capacity such as superoxide dismutase and catalase^75^.

Concentrations of nanocarriers were used at a dose of 10 μM, in accordance with previously published data from our group^26,45,52^. Free drug curcumin was shown to significantly increase viability of cells treated with varying concentrations of ethanol across all concentrations and time points. Curcumin free drug co-treatment at both 5 μM and 10 μM concentrations were able to completely ameliorate loss of viability caused by ethanol back to control levels in some cases. Across both time points, curcumin DSPE-PEG nanocarriers were able to protect to at least the same extent as the free drug against the ethanol induced loss of cell which suggests 3 h pre-treatment of 10 μM curcumin DSPE-PEG could be protective against oxidative stress and liver injury in ARLD. Curcumin DSPE-PEG nanocarriers were also able to protect against ROS production. The specific mechanisms of action behind the novel nanocarriers are complex however, it is thought that their effects are due to modulation of antioxidant signalling and mitochondrial function.

Previous research has suggested that treatment with curcumin may alter pathways associated with liver disease such as TGF-β1/Smad, JNK1/2-ROS, NF-κB as well as antioxidant signalling^76^. Curcumin mechanisms of action may also be due to the pentose-phosphate pathway. The pentose-phosphate pathway functions to supply NADPH which is required for conversion of oxidised GSH to reduced GSH via glutathione reductase^60,77^. Therefore, it is possible that the ability of entrapped curcumin to protect against ethanol may be due to their potential in increasing intracellular GSH. In support of this, we have previously used *N*-acetylcysteine formulations in a model of Parkinsons disease^26^, where a reduction in oxidative stress was observed. This data supports the therapeutic possibility of curcumin use in antioxidant therapy for ARLD, since ethanol is implicated in the loss of antioxidant capacity and increases in oxidative stress. Taken together, this data suggests that both free antioxidant compounds and entrapped compounds provide beneficial effects on protecting against oxidative stress in an ARLD model.

Whilst curcumin nanoformulations have shown promise in a number of in vitro and *in-vivo* studies^78,79^ this is now being translated into clinical investigations. For example, treatment of patients undergoing coronary elective angioplasty with curcumin nanoformulations showed lower indicators of oxidative injury^80^, whereas, protective effects with curcumin nanoformulations during cancer treatment has been demonstrated in a number of cancers^81^. Furthermore, there are ongoing clinical trials examining curcumin nanoformulations in colon cancer (NCT01294072). Despite this, there are a number of challenges that lie ahead. Whilst the dose of curcumin has been shown to be safe in a number of clinical studies, long term usage remains to be determined.

There are number of limitations in the current study. Whilst we only focussed on 350 mM alcohol treatment when studying curcumin nanoformulations, it would be useful to examine varying concentrations of alcohol from 100 mM combined with curcumin formulations. In addition, further examination of inflammatory pathways, including studying antioxidant levels such as GSH will provide further information into the mechanisms of protection provided by curcumin. Finally, we need to undertake *in-vitro* studies beyond three days. Future improvements include evaluation of other antioxidant based nanformulations, such as NAC, and translation of *in-vitro* findings to *in-vivo* models, and preclinical studies.

## Conclusion

Overall, this study observed that exposure to ethanol resulted in notable cellular toxicity, particularly pronounced at higher concentrations of ethanol. Additionally, ethanol treatment led to increases in the production of ROS and apoptosis, indicating a distinct correlation between alcohol consumption and levels of oxidative stress. The study further identified disruptions to mitochondrial regulation following alcohol treatment, including reductions in mitochondrial membrane potential. Consequently, mitochondrial dysregulation as well as mitochondrial damage caused by alcohol has the potential to impede cellular energy production and contribute to apoptosis. This study demonstrates for the first time that successful entrapment of curcumin into DSPE-PEG carriers provides protection against oxidative stress in a cellular model of ARLD. These results show that ethanol causes significant reduction in cell viability and increases ROS production. Treatment with carrier entrapped antioxidants was able to enhance or match the protective effects of the free drugs. This study demonstrates evidence for the use of nanoformulated antioxidants in the treatment of ARLD. However, further investigations are required to assess the ability of these formulations to protect against other parameters of ARLD.

## Supplementary Information

Supplementary Information 1.

Supplementary Information 2.

Supplementary Information 3.

Supplementary Information 4.

Supplementary Information 5.

## Data Availability

The datasets used and/or analysed during the current study are available from the corresponding author upon reasonable request.
