# Supplementary figures and images for: Oxidative stress in a cellular model of alcohol-related liver disease: protection using curcumin nanoformulations
Source: Sci Rep. 2025 Mar 5;15:7752. doi: 10.1038/s41598-025-91139-0 (PMC11882943; doi:10.1038/s41598-025-91139-0)

**A**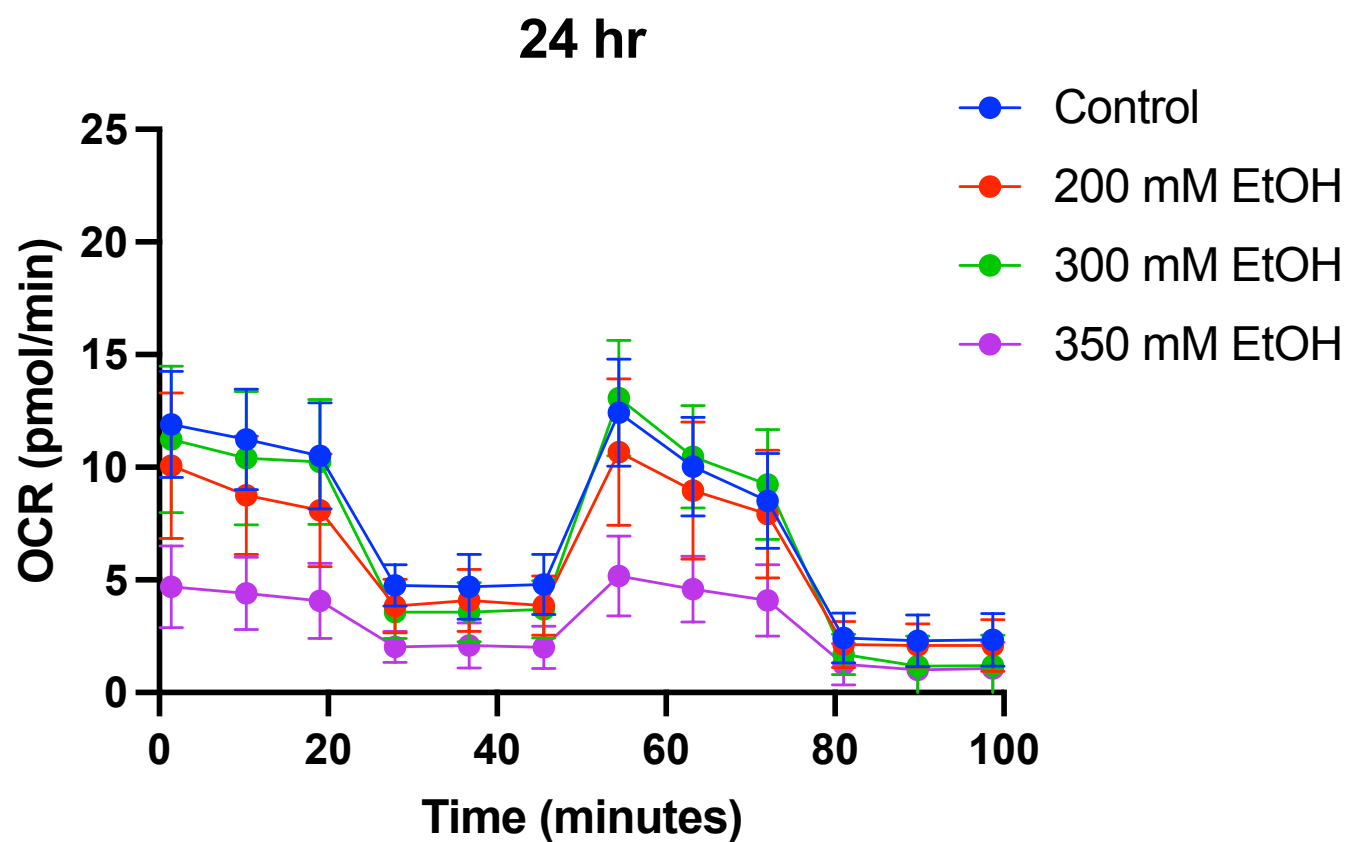**B**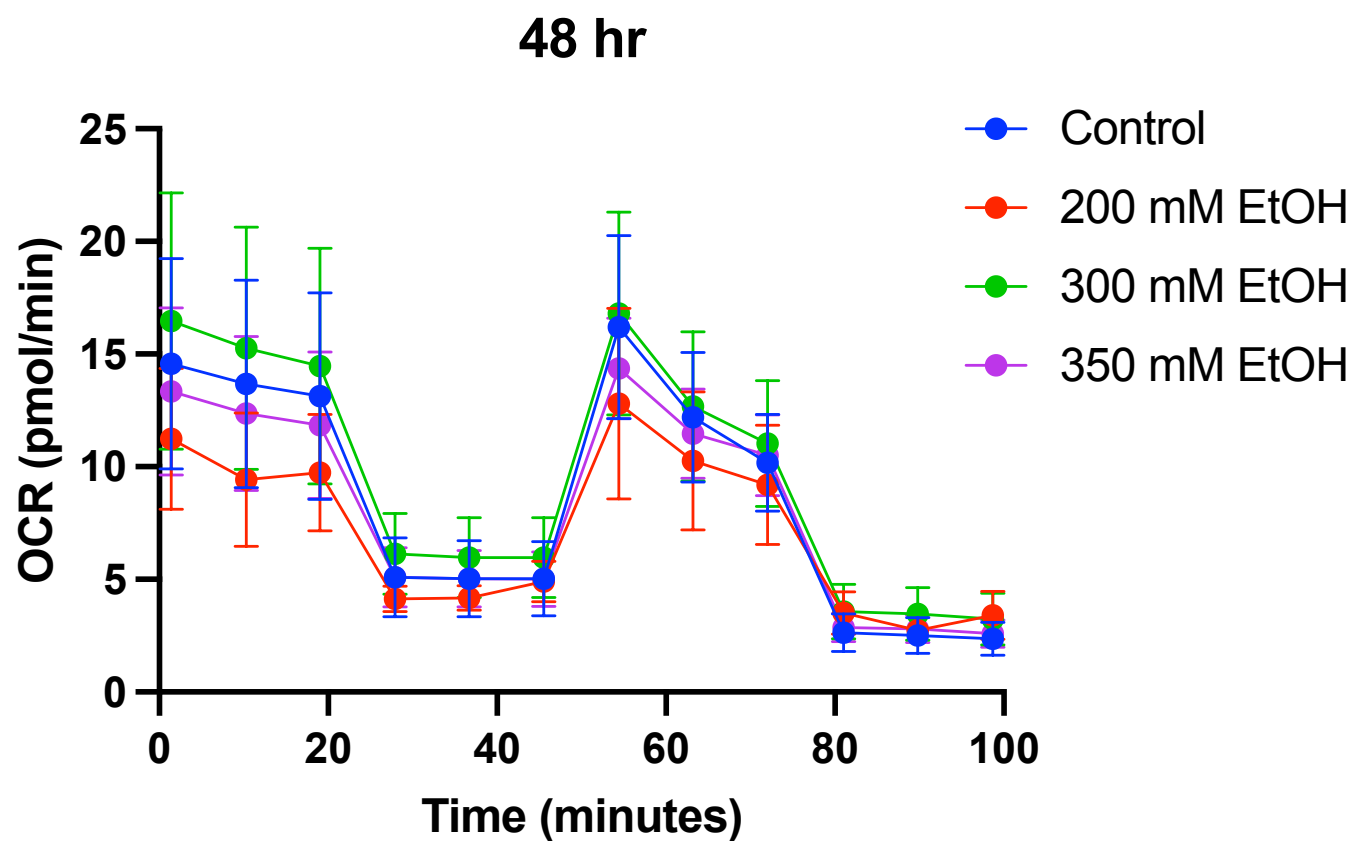

Supplement: Supplementary file 1 — Supplementary Information 1. [file 41598_2025_91139_MOESM1_ESM.pdf]

**A**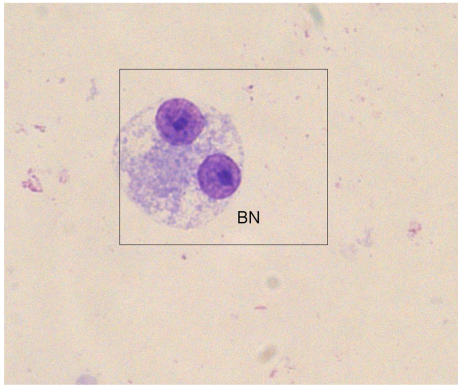**B**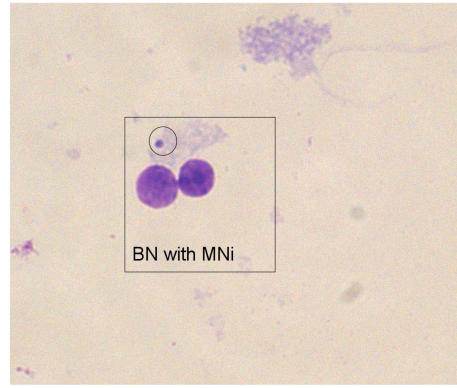**C**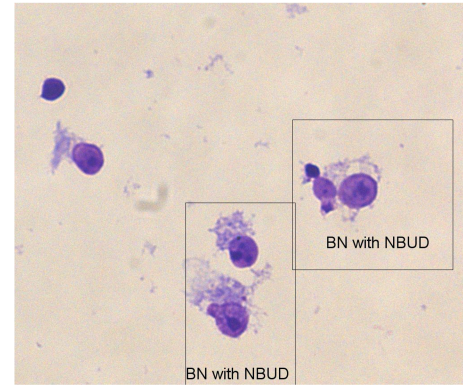**D**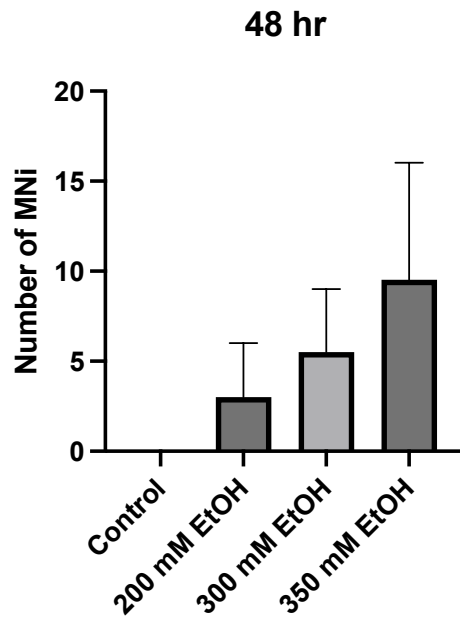**E**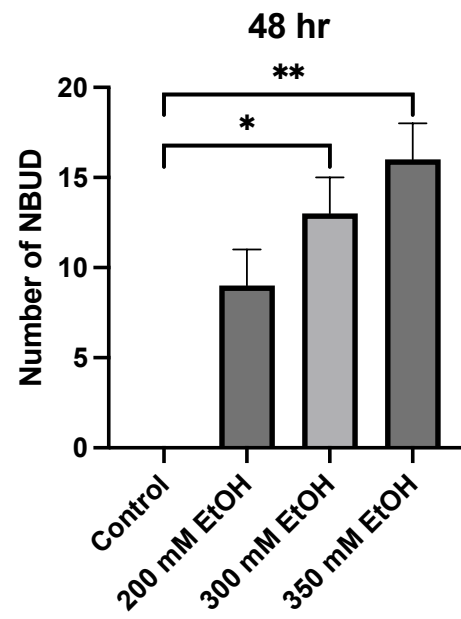**F**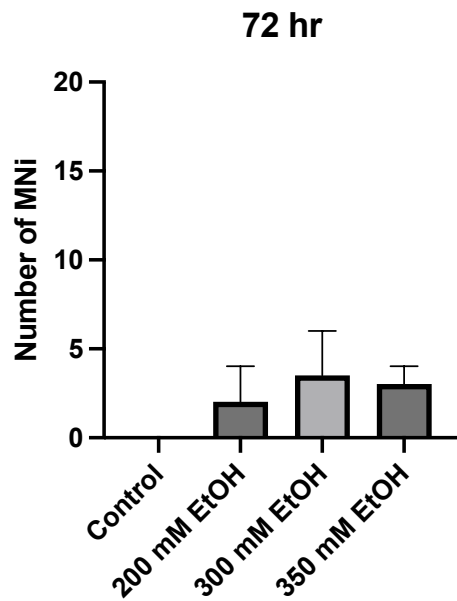**G**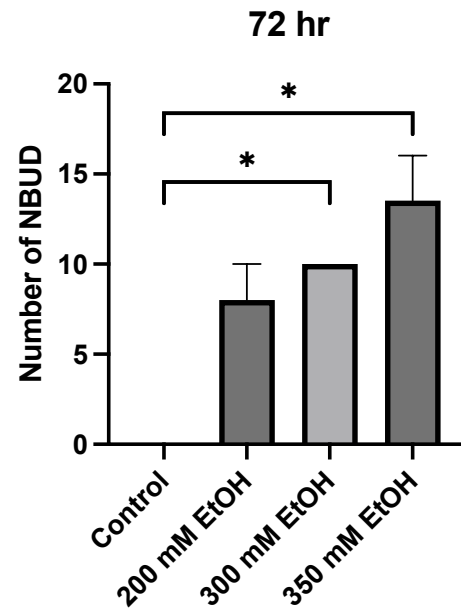

Supplement: Supplementary file 4 — Supplementary Information 4. [file 41598_2025_91139_MOESM4_ESM.pdf]
