# Supplementary material for: Oxidative stress in a cellular model of alcohol-related liver disease: protection using curcumin nanoformulations
Source: Sci Rep. 2025 Mar 5;15:7752. doi: 10.1038/s41598-025-91139-0 (PMC11882943; doi:10.1038/s41598-025-91139-0)
Supplement: Supplementary file 3 — Supplementary Information 3. [file 41598_2025_91139_MOESM3_ESM.pdf]

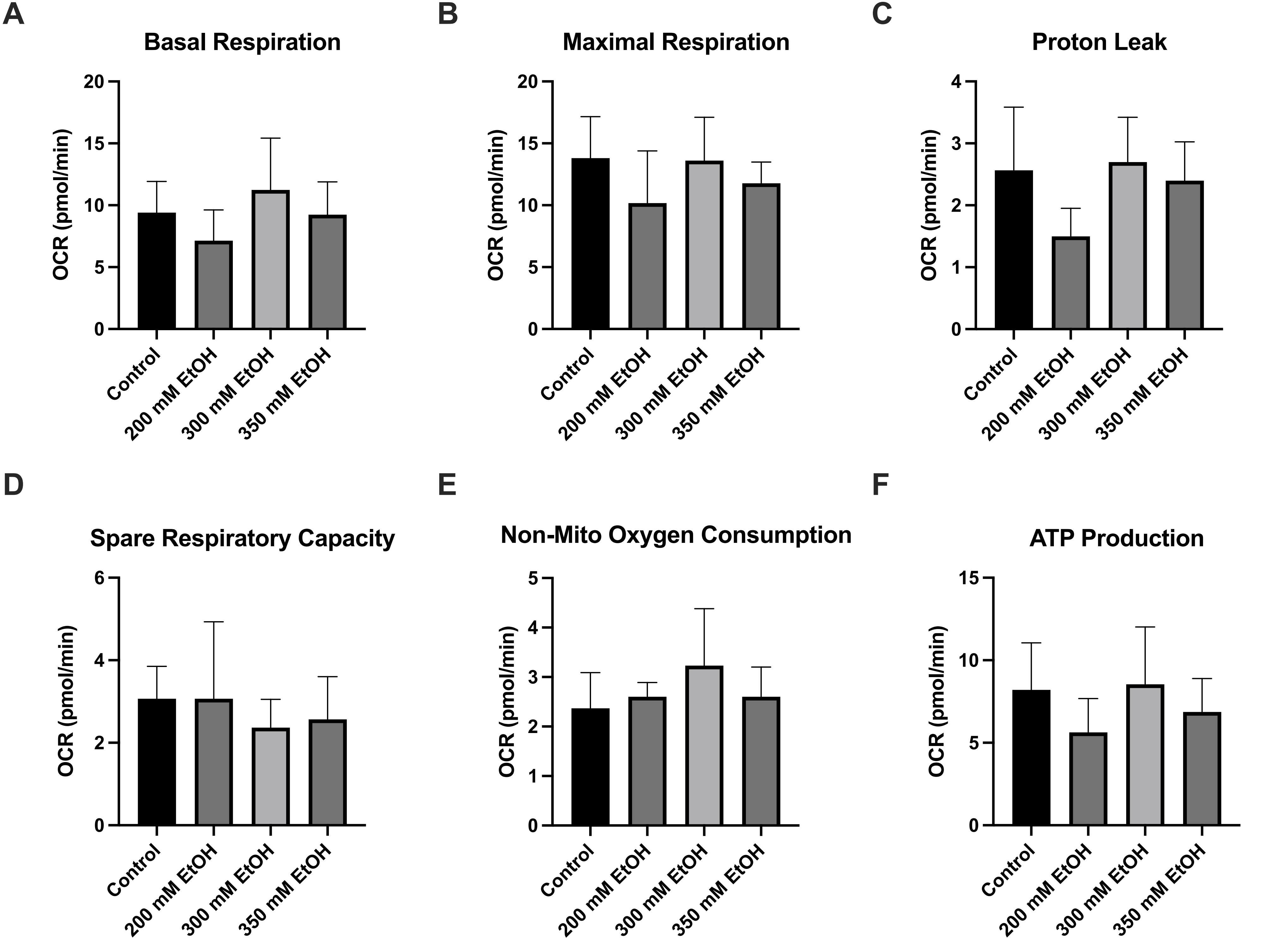
A
Basal Respiration
OCR (pmol/min)
0
5
10
15
20
Control
200 mM EtOH
300 mM EtOH
350 mM EtOH
B
Maximal Respiration
OCR (pmol/min)
0
5
10
15
20
Control
200 mM EtOH
300 mM EtOH
350 mM EtOH
C
Proton Leak
OCR (pmol/min)
0
1
2
3
4
Control
200 mM EtOH
300 mM EtOH
350 mM EtOH
D
Spare Respiratory Capacity
OCR (pmol/min)
0
2
4
6
Control
200 mM EtOH
300 mM EtOH
350 mM EtOH
E
Non-Mito Oxygen Consumption
OCR (pmol/min)
0
1
2
3
4
5
Control
200 mM EtOH
300 mM EtOH
350 mM EtOH
F
ATP Production
OCR (pmol/min)
0
5
10
15
Control
200 mM EtOH
300 mM EtOH
350 mM EtOH
