# Supplementary material for: Oxidative stress in a cellular model of alcohol-related liver disease: protection using curcumin nanoformulations
Source: Sci Rep. 2025 Mar 5;15:7752. doi: 10.1038/s41598-025-91139-0 (PMC11882943; doi:10.1038/s41598-025-91139-0)
Supplement: Supplementary file 5 — Supplementary Information 5. [file 41598_2025_91139_MOESM5_ESM.docx]

**SUPPLEMENTARY DATA**

**Supplementary Figure S1. Mitochondrial oxygen consumption rate (OCR) after ethanol exposure for (A) 24 hr and (B) 48 hr**. OCR was assessed over 48 hr using the Seahorse XF24 analyser. Results presented as mean of replicates ± SEM (n = 3).

Supplementary Figure S2. The effect of ethanol on mitochondrial respiration at 24 hr. (A) basal respiration, (B) maximal respiration, (C) proton leakage, (D) spare respiratory capacity, (E) non-mitochondrial oxygen consumption and (F) ATP production. OCR was assessed at 24-hr using the Seahorse XF24 analyser. Results presented as mean of replicates ± SEM (n = 3).

Supplementary Figure S3. The effect of ethanol on mitochondrial respiration at 48 hr. (A) basal respiration, (B) maximal respiration, (C) proton leakage, (D) spare respiratory capacity, (E) non-mitochondrial oxygen consumption and (F) ATP production. OCR was assessed at 24-hr using the Seahorse XF24 analyser. Results presented as mean of replicates ± SEM (n = 3).

**Supplementary Figure S4. Representative analysis of genome damage in response to ethanol.** (A) representative image of binucleated cells, (B) representative image of micronuclei, (C) representative image of nuclear budding, (D) number of micronuclei at 48 hr, (E) number of nuclear buds at 48 hr, (F) number of micronuclei at 72 hr and (G) number of nuclear buds at 72 hr. Cells were fixed and stained on slides using Gimsea. Data is presented as percentage difference from the control. Results presented as mean ± SEM (n = 2).
